# Mitigating methane emission from paddy soil with rice-straw biochar amendment under projected climate change

**DOI:** 10.1038/srep24731

**Published:** 2016-04-19

**Authors:** Xingguo Han, Xue Sun, Cheng Wang, Mengxiong Wu, Da Dong, Ting Zhong, Janice E. Thies, Weixiang Wu

**Affiliations:** 1 Institute of Environmental Science and Technology, Zhejiang University, Hangzhou 310058, PR China; 2 Provincial Key Laboratory for Water Pollution Control and Environmental Safety Zhejiang University, Hangzhou 310058, PR China; 3 Soil and Crop Sciences Section, School of Integrative Plant Science, Cornell University, Ithaca, NY, 14853, USA

## Abstract

Elevated global temperatures and increased concentrations of carbon dioxide (CO_2_) in the atmosphere associated with climate change will exert profound effects on rice cropping systems, particularly on their greenhouse gas emitting potential. Incorporating biochar into paddy soil has been shown previously to reduce methane (CH_4_) emission from paddy rice under ambient temperature and CO_2._ We examined the ability of rice straw-derived biochar to reduce CH_4_ emission from paddy soil under elevated temperature and CO_2_ concentrations expected in the future. Adding biochar to paddy soil reduced CH_4_ emission under ambient conditions and significantly reduced emissions by 39.5% (ranging from 185.4 mg kg^−1^ dry weight soil, dws season^−1^ to 112.2 mg kg^−1^ dws season^−1^) under simultaneously elevated temperature and CO_2_. Reduced CH_4_ release was mainly attributable to the decreased activity of methanogens along with the increased CH_4_ oxidation activity and *pmoA* gene abundance of methanotrophs. Our findings highlight the valuable services of biochar amendment for CH_4_ control from paddy soil in a future that will be shaped by climate change.

Climate change is unequivocal and inevitable. Since the 1950 s, average global mean surface temperature has increased by 0.72 °C and it is projected to further increase by 1.5 to 4.5 °C by the end of this century^1^. Atmospheric carbon dioxide (CO_2_) has also risen to 391 ppm at 2011, which exceeded the pre-industrial levels by about 40%, and is predicted to increase to between 421 and 936 ppm by 2100^1^. Projected increases in global temperature and atmospheric CO_2_ threaten future food security. It is well reported that crop yields will be influenced remarkably by interactions between elevated atmospheric CO_2_ and temperature^2^,^3^,^4^. Results of a meta-analysis on the response of rice yield to warming conditions (+0.8 °C to +6 °C) suggest that warming would significantly decrease yields by 14.6% for every 1 °C increase in temperature^5^.

Although many factors contribute to the warming trends observed, the main driver is generally attributed to increasing greenhouse gas (GHG) emissions. Methane (CH_4_) is the second most important GHG after CO_2_. It has over 25 times the global warming potential of CO_2_ over a 100-year forward prediction^6^ and is responsible for approximately 20% of the anthropogenic warming effect^7^. Rice cropping systems are considered to be among the major anthropogenic sources of CH_4_^8^. Estimates of the annual contribution of CH_4_ emissions from paddy soils range from 31 to 112 Tg y^−1^, accounting for 9–19% of total global CH_4_ emissions^8^,^9^. Worse still, a huge number of farmers have been pouring fertilizers and rice straws into paddy soil, both of which lead to increased emissions of CH_4_^10^,^11^,^12^ and thus aggravate climate warming.

Many studies have demonstrated that elevated atmospheric CO_2_ and temperature would further increase CH_4_ emission from paddy fields^9^,^13^,^14^,^15^. Meta-analyses of the effect of rising atmospheric CO_2_ and warming on CH_4_ emissions from rice paddies showed that increased CO_2_ levels of 460–780 ppm could stimulate CH_4_ emissions by over 40%^5^,^13^. However, reduced CH_4_ emissions from paddy soil have also been reported to occur with elevated temperature and CO_2_^16^,^17^,^18^. Regardless of the positive or negative effect of climate change, CH_4_ emission control from paddy soil is of critical importance now and in the future.

Biochar is the charcoal product resulting from the thermal decomposition of organic materials under a limited oxygen supply (pyrolysis)^19^. Biochar is not a homogeneous product, rather its properties vary according to the organic materials (feedstock) pyrolyzed and the time and temperature of pyrolysis. Biochar is a popular soil amendment intended to improve soil fertility by increasing soil nutrient retention^19^,^20^ and increasing soil water holding capacity thus enhancing primary productivity^21^. Biochar amendment is also promoted as a means to sequester stabilized carbon (C) in soil^22^,^23^,^24^. Previous studies have shown that amending paddy soils with biochar derived from rice straw can significantly decrease CH_4_ emissions by more than 80% compared to corresponding controls^25^,^26^,^27^. Decreased CH_4_ emissions were attributed mainly to the effects of biochar on soil physicochemical factors and changes in microbial communities, particularly decreases in the abundance and activity of the methanogens that produce CH_4_ and increases in the abundance and activity of the methanotrophs that oxidize it^26^,^27^,^28^,^29^. However, it remains unknown whether biochar can be used effectively to reduce CH_4_ emissions from rice cropping systems under projected changes in global temperature and atmospheric CO_2_ concentrations.

We investigated the effects of rice straw-derived biochar on CH_4_ emission from paddy soil under elevated temperature and CO_2_ through a chamber-scaled experiment. To provide adequate evidence and reveal the potential mechanism, we determined soil biochemical variables, the abundances of 16 S rRNA genes of methanogens, the abundances of the particulate methane monooxygenase genes (*pmoA*), and rice plant growth and yield. Results of this study will provide solid evidence that rice straw-derived biochar is an effective soil amendment for reducing CH_4_ emissions from paddy soils under projected climate change.

## Results

### Rice plant growth

**Figure 1:**
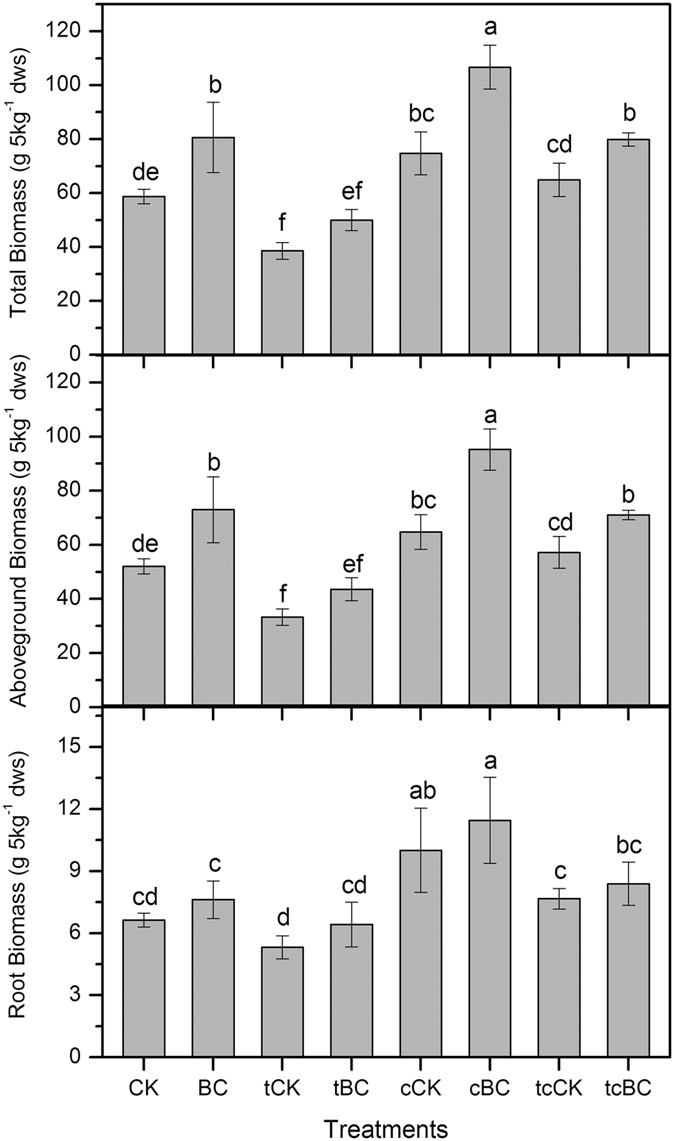
Total biomass, above-ground biomass (stems, leaves and grains) and root biomass of rice plants across all treatments. The values presented in the columns are mean ± standard deviations (n = 4). Different lowercase letters indicate significant differences between the eight treatments (*p* < 0.05). Rice plants were grown under ambient (CK, BC) or elevated temperature (tCK, tBC), or elevated CO_2_ (cCK, cBC), or simultaneously elevated temperature and CO_2_ (tcCK, tcBC). Paddy soil was either unamended (CK) or amended with biochar (BC) (2.5% w/w).

Paddy soil was either amended with biochar (BC treatments) or left unamended (CK treatments) and then planted with rice. Rice plants were grown under ambient (CK, BC) or elevated temperature (+3 °C, tCK, tBC), or elevated CO_2_ (700 ppm, cCK, cBC), or simultaneously elevated temperature and CO_2_ (+3 °C and 700 ppm, tcCK, tcBC). Elevated temperature alone (tCK) significantly (*p* < 0.05) reduced total and above-ground rice biomass respectively compared to the control (CK) (Fig. 1). Elevated CO_2_ alone (cCK) significantly (*p* < 0.05) promoted the total, above-ground and root biomass of rice plants grown than the corresponding control (CK). Biochar amendment under ambient (BC) and elevated CO_2_ (cBC) conditions increased the total and above-ground biomass of rice plants significantly (*p* < 0.05) compared to their corresponding controls (CK and cCK). Moreover, biochar addition in the simultaneously elevated temperature and CO_2_ system significantly (*p* < 0.05) increased the total and above-ground biomass of rice plants, respectively.

### CH_4_ emission patterns

**Figure 2:**
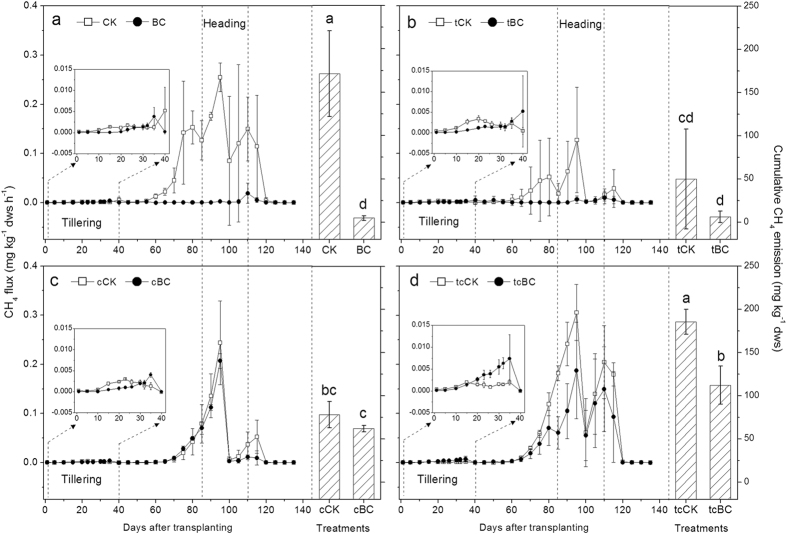
Seasonal variation of CH_4_ emission flux under different temperature and CO_2_ concentration with and without BC amendment (**a**) ambient; (**b**) elevated temperature; (**c**) elevated CO_2_; (**d**) elevated temperature and CO_2_ simultaneously) during the whole rice growing season, and the cumulative CH_4_ emissions in different treatments. The total cumulative methane emissions from 0 to 135 days are shown in Supplementary Fig. S1 in the “Supplementary Information”. Treatment designations of the cumulative CH_4_ emissions are showed below each column and different letters indicate significant differences between the eight treatments (*p* < 0.05). Treatment legend is given in Fig. 1.

There was a similar trend of CH_4_ emission flux across all treatments during the overall rice growing season with the peak occurring at the heading stage (Fig. 2). The CH_4_ emission flux in tCK and cCK at the heading stage was much lower compared to that in CK, but higher in tcCK. Biochar amendment, to some extent, reduced the CH_4_ emission flux from paddy soil under all experimental conditions. The cumulative CH_4_ emissions from the paddy soils during the overall rice growing season showed remarkable differences among all treatments (Fig. 2). In contrast to CK, the cumulative CH_4_ emissions from tCK and cCK were significantly lower (*p* < 0.05). No significant difference in the cumulative CH_4_ emissions was observed between tcCK and CK. Biochar addition reduced CH_4_ emissions under ambient conditions remarkably (*p* < 0.05), ranging from 171.2 mg kg^−1^ dry weight soil, dws to 4.8 mg kg^−1^ dws. The addition of biochar either under elevated temperature or elevated CO_2_ had no significant impact on the cumulative CH_4_ emissions. Nevertheless, the application of biochar played a notable role in reducing the cumulative CH_4_ emissionsfrom paddy soil under simultaneously elevated temperature and CO_2_ conditions (*p* < 0.05). There was a significantly (*p* < 0.05) lower cumulative CH_4_ emissions from tcBC (112.2 mg kg^−1^ dws) than that from tcCK (185.4 mg kg^−1^ dws).

### Soil methanogenic and CH_4_ oxidation activity

**Figure 3:**
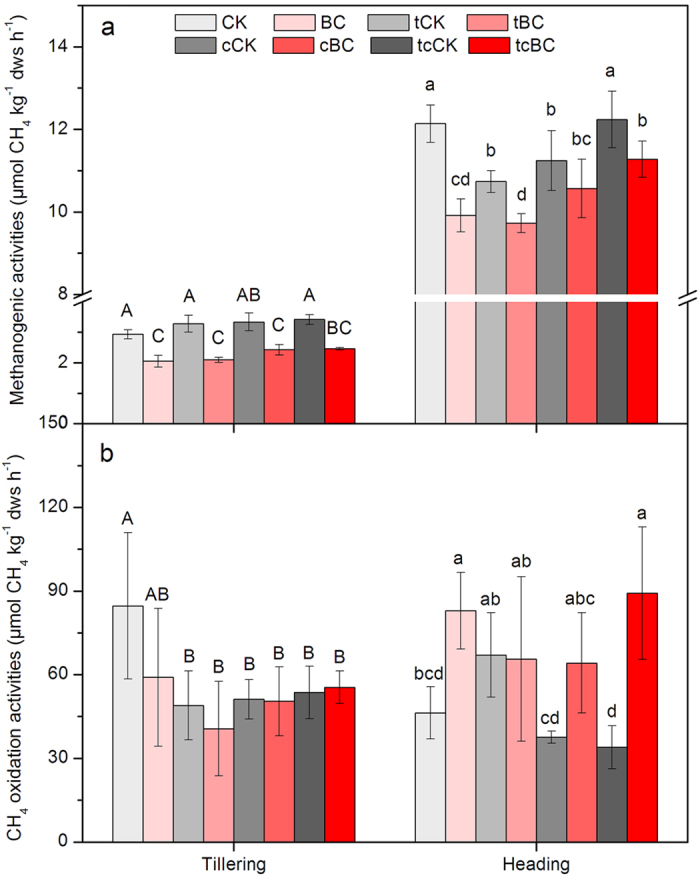
Methanogenic (**a**) and CH_4_ oxidation activity (**b**) in the rhizosphere soil at the tillering and heading stages in different treatments. Different letters indicate significant differences between the eight treatments at the same rice stage (*p* < 0.05). Treatment legend is given in Fig. 1.

Variations of methanogenic and CH_4_ oxidation activity in rhizosphere soils at the tillering and the heading stages are presented in Fig. 3. Although there were no significant differences in soil methanogenic activity among CK, tCK, cCK and tcCK at the tilling stage, significantly (*p* < 0.05) lower activity in tCK (10.7 μmol CH_4_ kg^−1^ dws h^−1^) and cCK (11.2 μmol CH_4_ kg^−1^ dws h^−1^) was detected at the heading stage as compared with that in CK (12.1 μmol CH_4_ kg^−1^ dws h^−1^) (Fig. 3a). The addition of biochar resulted in a significant reduction of methanogenic activity both at the tillering and heading stage under all conditions tested (*p* < 0.05), with the exception of the elevated CO_2_ at the heading phase (Fig. 3a). The soil CH_4_ oxidation activity in CK was much higher at the tilling stage in comparison with any other treatments (Fig. 3b). At the heading stage, despite no significant differences in the soil CH_4_ oxidation activity of tCK, cCK or tcCK in contrast to that of CK, biochar addition exerted a marked influence on the soil CH_4_ oxidation potential under different conditions. The CH_4_ oxidation activity in the BC and tcBC treatments was increased by 79.0% and 162.3% as compared to their corresponding controls (CK, tcCK) (*p* < 0.05) (Fig. 3b).

### Abundances of methanogenic archaeal 16 S rRNA genes and methanotrophic bacterial *pmoA* genes

**Figure 4:**
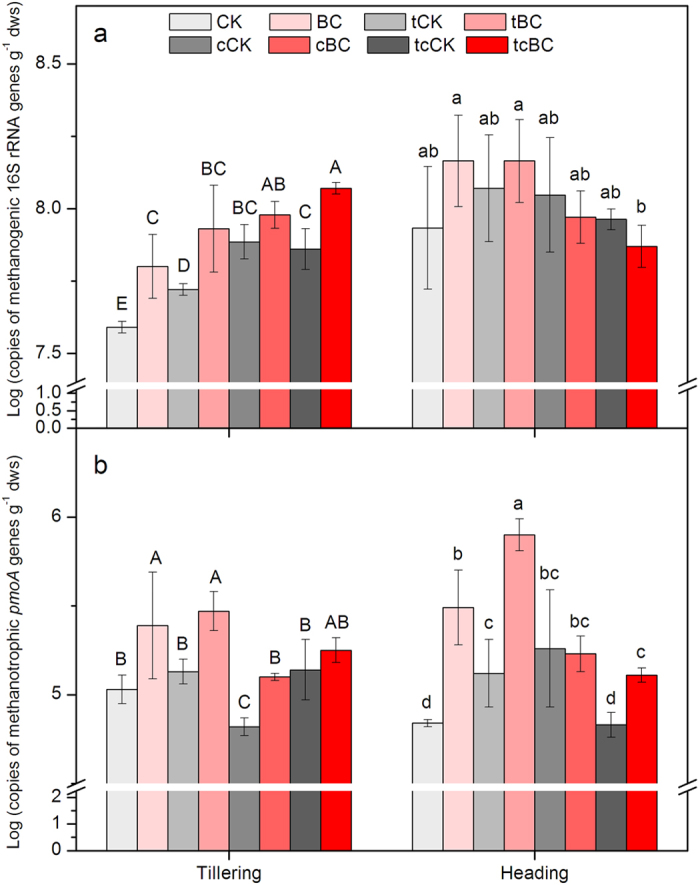
Abundance of methanogenic 16 S rRNA genes (**a**) and methanotrophic *pmoA* genes (**b**) in the rhizosphere soil at the tillering and heading stages in different treatments. Different letters indicate significant differences between the eight treatments at the same rice stage (*p* < 0.05). Treatment legend is given in Fig. 1.

Responses of the copy numbers of methanogenic archaeal 16 S rRNA genes in paddy soil to biochar addition, elevated temperature, elevated CO_2_, and simultaneously elevated temperature and CO_2_ are shown in Fig. 4a. At the tillering stage, methanogenic archaeal 16 S rRNA genes abundance from tCK, cCK or tcCK was significantly (*p* < 0.05) higher than that from CK. Biochar amendment significantly (*p* < 0.05) increased the copy numbers of 16 S rRNA genes for methanogens at the tilling stage, with the exception of elevated CO_2_. There were no significant differences in methanogenic archaeal 16 S rRNA genes abundance among paddy soils between BC addition and non-amended treatments at the heading stage.

Changes in the copies of methanotrophic *pmoA* gene in different paddy soils are showed in Fig. 4b. At the tillering stage, only elevated CO_2_ resulted in significant reduction of soil methanotrophic *pmoA* gene abundance (*p* < 0.05). Biochar amendment significantly (*p* < 0.05) increased the abundance of methanotrophic *pmoA* gene under ambient, elevated temperature, and elevated CO_2_ condition. At the heading stage, copy numbers of methanotrophic *pmoA* gene from tCK and cCK were significantly (*p* < 0.05) higher than that from CK. Compared with their corresponding control, biochar addition led to a significant increase in the methanotrophic *pmoA* gene abundance (*p* < 0.05) except under the elevated CO_2_ condition. In contrast, the copy number of methanotrophic *pmoA* gene from tcBC (1.30 × 10^5^ copies g^−1^ dws) was significantly (*p* < 0.05) lower than that from BC (3.38 × 10^5^ copies g^−1^ dws).

## Discussion

This is the first study to investigate the role of biochar in mitigating CH_4_ emission from paddy soil under elevated temperature and CO_2_ condition. It is imperative to better understand the emission of CH_4_ influenced by different temperature and CO_2_ concentration, as well as to address and elucidate the mechanistic effects of biochar amendment on CH_4_ emission from paddy soil under the predicted climate change.

In this study, variations of temperature and CO_2_ concentration had different influences on CH_4_ emissions during the rice growing season (Fig. 2). Our results revealed that CH_4_ emissions were reduced under elevated temperature or elevated CO_2_ alone, where cumulative CH_4_ emissions were reduced by 70.9% and 54.4%, respectively, compared with those treatments under ambient temperature and CO_2_. However, elevating temperature and CO_2_ simultaneously did not exert any significant effects on the cumulative CH_4_ emissions, rather increased them by 8.3%. The different responses of CH_4_ emission to environmental factors might be due to the variations in rice plant growth and subsequent effects on CH_4_ production. Elevated temperature significantly reduced the total and above-ground biomass of rice plants (Fig. 1). Baker *et al.*^30^ also reported the reduced rice yield under elevated temperatures. Since 80–90% of CH_4_ released into the atmosphere was rice plant-mediated through the well-developed aerenchyma^31^,^32^, inhibited rice growth caused by elevated temperature can partially reduce the emissions of CH_4_ through rice plants^17^. The optimal temperature of most mesophilic methanogens is about 35 °C, with methanogenic activity showing a decreasing trend when above this temperature^33^,^34^,^35^. This is one of the causes that in our study, elevated temperature significantly decreased soil methanogenic activity at rice heading stage (Fig. 3a), and then weakened CH_4_ emissions. On the other hand, CO_2_ enrichment strikingly promoted the biomass of rice plants (Fig. 1), which could bring more available oxygen to the rhizosphere soil. These impacts led to the decreased CH_4_ emissions by depressing methanogenesis and accelerating the soil methanotrophic growth (Figs 3a and 4b). It was well supported by the results of Schrope *et al.*^17^ and Inubushi *et al.*^36^, who reported that CO_2_ enrichment reduced CH_4_ production and promoted CH_4_ oxidation through the benefit of increased oxygen delivery through rice plants. In addition, rice biomass was similar in the control (CK) and in soil with simultaneously elevated temperature and CO_2_ (tcCK) (Fig. 1). One possibility would be that CO_2_ enrichment weakened the negative effects of elevated temperature on rice plants growth, providing a certain amount of substrates for CH_4_ production. However, the observed results could not come to such an effect. Further research is required before conclusions can arrive. Increases in atmospheric temperature and CO_2_ concentration turned out to be driving forces for CH_4_ emission from paddy soil (Fig. 2). Thus, CH_4_ control from paddy soil needs to be paid more attention in the future due to the predicted rise of both temperature und CO_2_.

Our results confirmed that CH_4_ emissions in paddy soil under ambient condition were reduced significantly by the application of biochar. Biochar amendment did significantly reduce the cumulative CH_4_ emissions by 97.2% compared to the control (Fig. 2a). The addition of biochar under ambient conditions attenuated the methanogenic activity remarkably at both the tillering and the heading stages, and improved methanotrophic *pmoA* gene abundance and potential activity at the heading stage (Figs 3a and 4b). These findings are similar to the results of many previous studies, which reported that biochar amendment could make the rhizosphere soil favorable for methanotrophs but unfavorable for methanogens^25^,^26^,^27^. Therefore, it was the stimulated methanotrophic activity and inhibited methanogenic activity caused by biochar application that led to the declined CH_4_ emissions in ambient system. These results verify that the application of rice straw biochar not only stimulate rice plant productivity^37^,^38^, but also suppress CH_4_ emissions from paddy soil.

**Figure 5:**
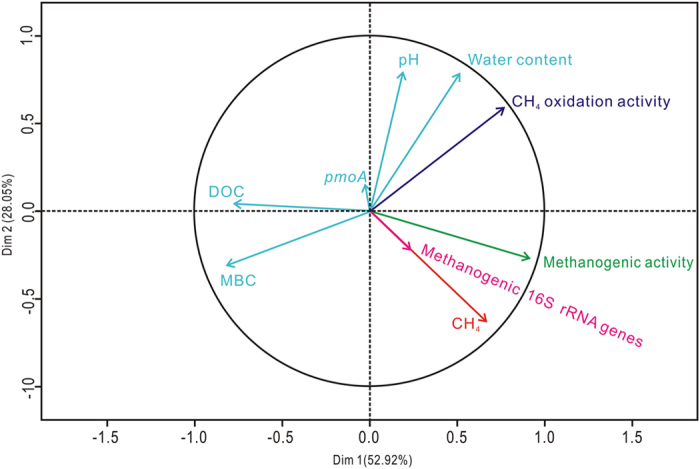
The correlation circle of CH_4_ emission and biochemical and microbial characteristics during the rice growing season. Dim 1 and Dim 2 represent the ratio of respective index in the whole system.

Interestingly, biochar amendment also significantly decreased CH_4_ emission from paddy soil under simultaneously elevated temperature and CO_2_ condition. Compared to the corresponding control (tcCK), the cumulative CH_4_ emissions in tcBC were reduced by 39.5% (Fig. 2d). Based on the role of biochar in decreasing CH_4_ emission under ambient environmental conditions, we hypothesized that biochar addition would have notable influence on CH_4_ production and oxidation under the combined condition. As was expected, methanogenic activity decreased and CH_4_ oxidation potential increased when biochar was applied in simultaneously elevated temperature and CO_2_ system at the heading stage (Fig. 3). Spearman correlations and the correlation circle were calculated to compare the CH_4_ emission rates with biochemical and microbial gene data. We observed a significantly positive correlation between CH_4_ emission rate and methanogenic activity (rho = 0.500, *p* < 0.01) and CH_4_ oxidation activity (rho = 0.533, *p* < 0.01) (Fig. 5; Supplementary Table S1). This confirms the important role that methanogenic activity and CH_4_ oxidation activity have in controlling CH_4_ emission from paddy soil.

It is well-known that variations of biochemical and microbial parameters can cause different CH_4_ fluxes by influencing CH_4_ production and oxidation processes. Soil CH_4_ production is affected by the availability of labile carbon substrates^39^. Soil dissolved organic carbon (DOC) contributes to a great deal of carbon sources for methanogenic growth^40^. A decrease of soil DOC content from tcBC compared to tcCK at the heading time could explain the reduced CH_4_ emissions observed due to the decreased methanogenic activity (Supplementary Fig. S2a). As is proved, absorption of soil organic carbon onto biochar particles may have reduced substrate availability for CH_4_ production^40^. Liu *et al.*^25^ also illustrated that biochar amendment significantly reduced the soil methanogenic activity and CH_4_ emissions from paddy soil, mainly benefiting from the lack of substrate availability for methanogens. Meanwhile, we observed a greater concentration of microbial biomass carbon (MBC) in tcBC at the heading time, which suggests a faster succession of microorganisms by consuming easily available soil organic carbon (Supplementary Fig. S2b). This may then have retarded CH_4_ production and methanogenic activity by forming a more recalcitrant organic carbon pool^41^. Moreover, biochar addition under simultaneously elevated temperature and CO_2_ condition significantly promoted rice plants growth by improving the total and above-ground biomass (Fig. 1). The stimulated rice plants could bring more oxygen to the aerenchyma tissues of rhizosphere^28^,^42^, thus inhibiting methanogenic activity and increasing methanotrophic activities (Fig. 3). Some studies also demonstrated that the high porosity and large surface area of biochar may enhance the adsorption of CH_4_^25^,^28^, providing substrates for methanotrophs and thus reducing CH_4_ emissions.

Furthermore, a significantly positive correlation of CH_4_ oxidation activity and soil water content (rho = 0.542, *p* < 0.01), and pH value (rho = 0.439, *p* < 0.05) indicated the important role of soil moisture and pH in affecting soil CH_4_ oxidation (Fig. 5; Supplementary Table S1). Soil water content increased considerably in tcBC in comparison to that in tcCK at the heading time (Supplementary Fig. S3). This broadened the optimum range of water content for methanotrophy^43^,^44^, and then stimulated CH_4_ oxidation activity (Fig. 3b). Studies have noted that the highly porous structure of biochar could increase water holding capacity, and thus increase CH_4_ oxidation by restricting soil moisture fluctuations^40^,^45^,^46^. Schnell *et al.*^47^ also reported that CH_4_ uptake rates would increase with increasing water content. Moreover, methanotrophs are usually sensitive to the fluctuation of soil pH values^39^. Our results showed that biochar amendment could significantly increase the pH values ranging from 5.55 ± 0.10–5.71 ± 0.01 under simultaneously elevated temperature and CO_2_ condition at the heading stage (Supplementary Fig. S4). The increased pH was favorable for methanotrophs (the optimal pH value is 6.0–7.0)^48^, promoting methanotrophic potential (Fig. 3b).

The CH_4_ emission rate displayed significant negative correlations with the abundance of methanotrophic *pmoA* gene (rho = −0.558, *p* < 0.05) (Supplementary Table S1). In simultaneously elevated temperature and CO_2_ system, biochar addition improved the copy numbers of methanotrophic *pmoA* gene significantly, stimulating methanotrophic growth at the heading stage (Fig. 4b). As a result of the apparently promoted methanotrophic *pmoA* gene abundance, CH_4_ oxidation activity was enhanced greatly (Fig. 3b). The observed increase in the methanotrophic *pmoA* gene abundance might benefit from the abundant CH_4_ as the only C substrate for methanotrophs^26^, as well as from the oxygen condition^40^ and living habitat^49^ supplied by biochar addition. Our results were similar to Feng *et al.*^26^, who also showed that biochar addition could significantly promote methanotrophic growth with the increased abundances of *pmoA* gene in paddy soil, explaining the reduced CH_4_ emissions. This study demonstrated that biochar incorporation resulted in the significant increases in rice biomass, pH, moisture and methanotrophic *pmoA* gene abundance, and decrease in labile organic carbon. These variations would inhibit CH_4_ production and promote CH_4_ oxidation, and thereby lower CH_4_ emission under the combined condition.

The community structure and composition of methanogens and methanotrophs could exert great effects on CH_4_ production and oxidation^33^,^39^. Potential activity and the copy numbers of key genes observed in this study could not represent the structure and metabolism difference of methanogens and methanotrophs communities, during the heading time, although an evidently higher methanogenic activity and less stimulatory methanotrophic growth in tcBC statistically explained parts of the difference of CH_4_ emission under ambient and the combined conditions (Figs 3a and 4b). Thus, further research to assess the environmental functions and structures of methanogens and methanotrophs responsible in different systems is highly desirable. These findings suggested that although the effect of biochar addition on CH_4_ mitigation was weakened, it could also assist in making paddy soil a great CH_4_ sink under the combined condition (Fig. 2). It will provide valuable rice ecosystem services, which become increasingly important for CH_4_ control from paddy soil in the projected warming climate.

In conclusion, our findings not only confirmed that biochar amendment decreased CH_4_ emissions from paddy soil under ambient condition, but also, for the first time, demonstrated that biochar addition did significantly reduce CH_4_ emissions by 39.5% from paddy soil under simultaneously elevated temperature and CO_2_ conditions. The reduced CH_4_ emissions were mainly due to the reduced CH_4_ production and release as a result of the inhibition to methanogens and promotion to methanotrophs, which were caused by changes in biochemical and microbial characteristics with biochar addition. These results imply that biochar amendment into paddy soil can be an effective strategy for CH_4_ mitigation under the elevated temperature and CO_2_ condition. In addition, it is relevant as our earth is predicted to become warmer and highlights the value of biochar in assisting with slowing down the greenhouse effect caused by CH_4_ emission from paddy soil. Moreover, it will pave a way for human beings to curb the inevitably warming climate by adopting biochar to reduce the anthropogenic CH_4_ emissions. Nevertheless, the variation of structure and composition in functional methanogens and methanotrophs communities is still unclear. Further research about the effects of biochar amendment on CH_4_ mitigation from paddy soil needs to elucidate the microbial mechanism of CH_4_ release under simultaneously elevated temperature and CO_2_ condition.

## Methods

### Soil and Biochar

Paddy soil (0–15 cm) used in this study was collected from a traditional, representative rice field in the Yuhang District (119.5°E, 30.2°N), Hangzhou, Zhejiang Province, China. The soil was air-dried, ground and sieved through a 2 mm mesh screen prior to use. Rice straw-derived biochar was produced through slow pyrolysis of rice straw at 500 °C with a mean residence time of 3 h. The pH, electrical conductivity (EC), total carbon (TC) total nitrogen (TN), bulk density and cation exchange capacity (CEC) of the soil and biochar were measured and are given in Table 1.

### Microcosms

Microcosm studies were carried out at the Agricultural Experiment Station of Zhejiang University, Hangzhou, China, in a growth chamber system consisting of four 1.85 × 0.86 × 1.95 m (length × width × height) growth chambers, which could control the interior CO_2_ concentration, air temperature, lighting time and relative humidity automatically.

Biochar amendment treatments (BC) were applied at a rate of 2.5% (w/w) and mixed homogenously into the soil. A control treatment (CK) without biochar addition was established for comparison. Air temperature was set to approximately follow the ambient air temperature of April to August in Hangzhou city, China for the control treatments and +3 °C for the elevated temperature treatments (Supplementary Table S2). Lighting time was set near to the ambient condition (Supplementary Table S2). The ambient CO_2_ concentration was kept at 390 ± 10 ppm and the elevated CO_2_ was set at 700 ± 10 ppm. The eight treatment combinations were: CK and BC at ambient temperature and ambient CO_2_; the two soil treatments under elevated temperature and ambient CO_2_ (tCK and tBC); the two soil treatments under ambient temperature and elevated CO_2_ (cCK and cBC); and the two soil treatments under elevated temperature and elevated CO_2_ (tcCK and tcBC). Each treatment was replicated four times. Cylindrical polyvinylchloride pots (30 cm high and 20 cm inner diameter), equipped with a water tank (5 cm high) at the top for containing the gas sampling chamber and water seals, were filled with a 5 kg soil (CK) or a total 5 kg mass of soil and biochar.

All treatments were pre-incubated by flooding the soil with deionized water by 2–3 cm above the soil and placing them into the growth chambers for ten days before rice transplanting (*Oryza sativa* L). The pots were then randomly arranged at regular intervals to take account of subtle differences in light, temperature and CO_2_ within each chamber. Flooded conditions were maintained throughout the main rice growing stages, and then drained when the rice grew during the tillering stage (30 days after transplanting, 30 DAT). The draining ended at 40 DAT and the flooded conditions would stay thereafter. The basal fertilizers, urea-[CO(NH_2_)_2_] (60 mg N kg^−1^), and phosphate and potassic fertilizer-KH_2_PO_4_ and KCl (100 mg P_2_O_5_ kg^−1^ and 100 mg K_2_O kg^−1^) were applied and mixed homogenously at the pre-incubation time of treatments in chambers. Nitrogen fertilizer (urea) was top-dressed at early tillering phase (18 DAT) (45 mg N kg^−1^) and at the early heading phase (82 DAT) (45 mg N kg^−1^). Relative humidity was kept at 65 ± 2%.

### CH_4_ flux measurement

The CH_4_ flux was determined by the closed chamber method^50^. Details about the gas sampling procedure, structure of chamber and the measurement of CH_4_ concentration are presented in the “Supplementary Information”.

### Physicochemical analysis

During the pivotal rice growth phases - tillering (23 DAT) and heading (90 DAT) - rhizosphere soil samples were collected. Samples for molecular analysis and physicochemical measurements were kept at −70 °C and 4 °C, respectively.

Microbial biomass C (MBC), determined by the fumigation-extraction method^51^, and soil dissolved organic C (DOC), were both determined by an automated total organic C (TOC) Analyzer (Multi N/C 2100, Jena, Germany). Details about the measurement procedure of MBC and DOC are presented in the “Supplementary Information”. Soil pH was measured after suspending soil in water (1:2.5 w/w).

The methanogenic activity of paddy soils was measured in triplicate, by using fresh soil samples (10 g) mixed with 0.2 mmol oxygen-free sterile glucose solution in 100 ml serum bottle. The bottles were flushed with O_2_-free N_2_ for 3 min sealed with butyl rubber lids and aluminum crowns, and incubated at 28 °C for 24 h. CH_4_ contained in the headspace of the serum bottle was determined by gas chromatography. The methanogenic activity was expressed as micromoles of CH_4_ per kilogram of dry weight soil (dws) per hour^52^.

Soil CH_4_ oxidation activity was analyzed according to the method applied by Hanson^53^. Triplicate fresh soil samples (10 g) were placed in 100 mL serum bottles, sealed with butyl rubber lids and aluminum crowns. Each bottle was then injected with 5 mL of highly pure CH_4_ and incubated without light at 28 °C for 8 h. Empty but CH_4_-amended bottles were set as controls. CH_4_ in the headspace of the serum bottles was measured by using gas chromatography. CH_4_ oxidation activity was expressed as consumed micromoles of CH_4_ per kilogram of dry weight soil per hour^54^.

Rice biomass. After the rice grains were harvested, the above-ground parts of the rice plants were cut above 2 cm from the soil surface and removed. The rice roots in the pot were gently and thoroughly washed with water. The aboveground parts and roots of the rice plants were then oven-dried at 80 °C for 72 h to measure dry matter weight^55^.

### DNA extraction and quantification of functional microbial communities

For each sample at the rice tillering and heading stages, 0.5 g soil was used for DNA extraction with a FastDNA^®^ SPIN Kit for soil (MP Biomedical, LLC, OH, USA) according to the manufacturer’s instructions. The extracted soil DNA was then dissolved in 80 mL tris-EDTA (TE) buffer, stored at −20 °C until further use.

Quantitative PCR (qPCR) was used to estimate the abundances of methanogenic archaeal 16 S rRNA genes and *pmoA*, the functional gene encoding the key enzyme involved in methane oxidation (particulate methane monooxygenase) using the primer pairs 0357 F/0691 R^56^ and A189f/mb661r^57^, respectively. The quantification was based on the intensity of SYBR Green dye fluorescence. The PCR reaction mixture consisted of 2 μL of template DNA, 0.1 μmol L^−1^ of each primer, 1 × SYBR Premix EX Taq (Perfect Real Time) premix reagent and ultrapure DNase/RNase-free water (ddH_2_O) to a final volume of 20 μL. The primers and thermal cycling used for each reaction are given in “(Supplementary Table S3)”. Reactions were performed in triplicate in a Bio-Rad CFX1000 Thermal Cycler, and ddH_2_O was used as a negative control template. The gene copy numbers were calculated according to the method of Wang *et al.*^58^. Standard curves were obtained using purified plasmids of a 16 S rRNA gene of methanogens and *pmoA* gene clones.

### Statistical analyses

Treatment means and standard deviation (SD) were calculated. SPSS 20.0 statistical software package (SPSS Inc., Chicago, IL, USA) was used to determine significant difference among different treatments and correlation analysis. Significance of differences between groups was determined by analysis of variance *LSD* test. *p*-value < 0.05 was considered statistically significant. The correlation circle between CH_4_ emissions and other indexes was analyzed by the MFA (Multiple Factor Analysis) method through R software at http://www.R-project.org (R Development Core Team, 2014) from where it can be freely downloaded.

## Additional Information

**How to cite this article**: Han, X. *et al.* Mitigating methane emission from paddy soil with rice-straw biochar amendment under projected climate change. *Sci. Rep.* **6**, 24731; doi: 10.1038/srep24731 (2016).

## Supplementary Material

Supplementary Information

## Figures and Tables

**Table 1:** Selected soil and biochar physico-chemical parameters.

	Soil	Biochar
pH	5.09 (1:2.5H_2_O)	8.88 (1:10H_2_O)
EC(ms cm^−1^)	0.07	0.61
TC(%)	2.21	51.18
TN(%)	0.27	1.42
Bulk density(g cm^−3^)	n.d.	0.125
CEC(cmol kg^−1^)	n.d.	44.7
BET surface area(m^2^ g^−1^)	n.d.	75.5

n.d. not determined.
