# Supplementary material for: Mitigating methane emission from paddy soil with rice-straw biochar amendment under projected climate change
Source: Sci Rep. 2016 Apr 19;6:24731. doi: 10.1038/srep24731 (PMC4835783; doi:10.1038/srep24731)
Supplement: Supplementary Information [file srep24731-s1.doc]

# Supplementary Information

# Mitigating methane emission from paddy soil with rice-straw biochar amendment under projected climate change

Xingguo Han^1,2^†, Xue Sun^1,2^†, Cheng Wang^1,2^, Mengxiong Wu^1,2^, Da Dong^1,2^, Ting Zhong^1,2^, Janice E. Thies^3^ & Weixiang Wu^1,2^*

1. Institute of Environmental Science and Technology, Zhejiang University, Hangzhou 310058, PR China

2. Provincial Key Laboratory for Water Pollution Control and Environmental Safety.

3. Soil and Crop Sciences Section, School of Integrative Plant Science, Cornell University, Ithaca, NY, 14853, USA

* Corresponding Author (email: [weixiang@zju.edu.cn](mailto:weixiang@zju.edu.cn)).

† These authors contributed equally to this work.

Address: Institute of Environmental Science and Technology, Zhejiang University, 866 Yuhangtang Road, Hangzhou 310058, China

Tel: +86-571-88982020

Fax: +86-571-88902020


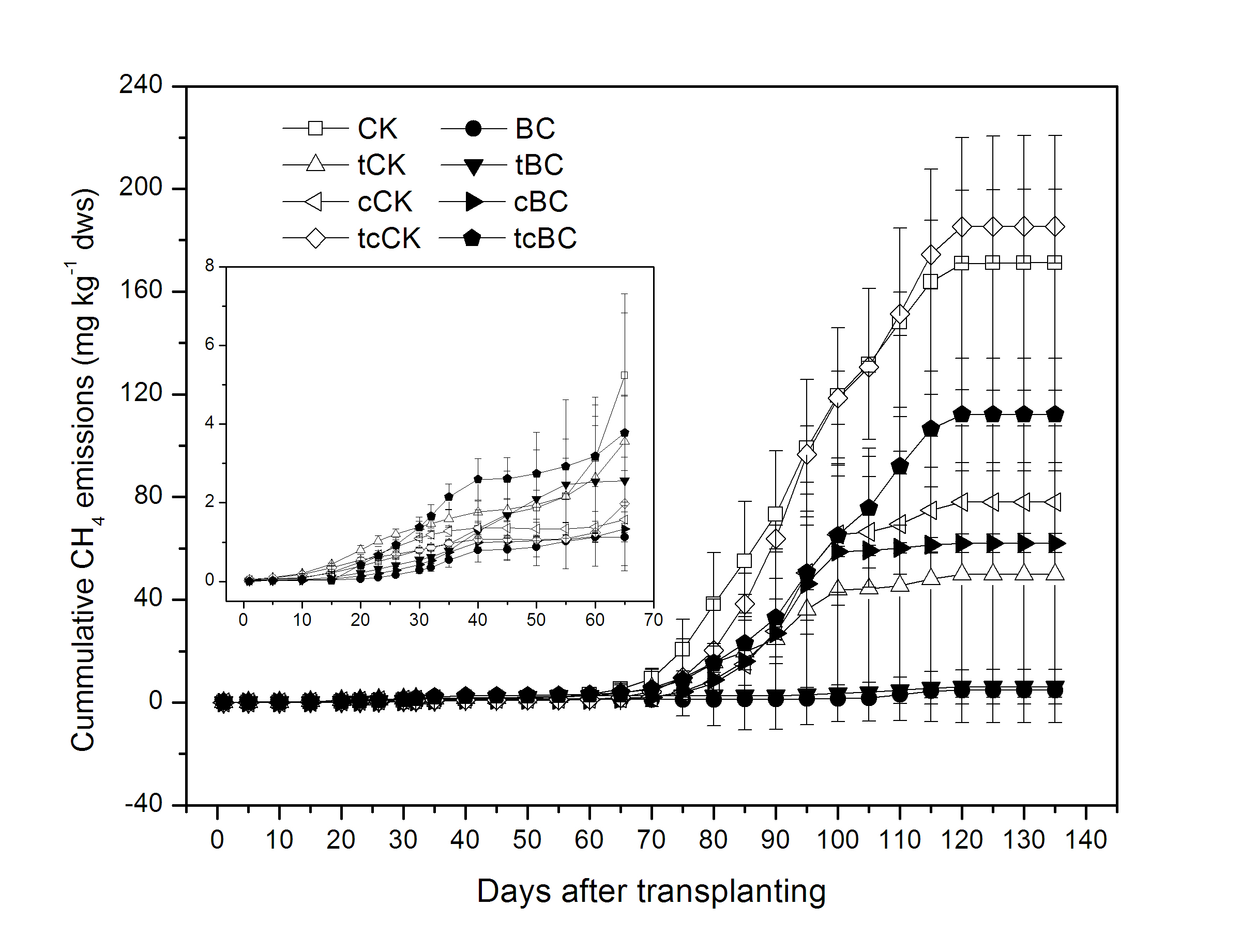


**Supplementary Figure S1. The total cumulative CH_4_ emissions in different treatments from 0 to 135 days after transplanting.** Treatment legend is given in Figure 1.

**Supplementary Figure S2. Soil dissolved organic C (DOC) (a) and microbial biomass carbon (MBC) (b) contents in different treatments at the rice tillering and heading stage.** Significant differences of different treatments at the same rice growing stage are showed by different letters over error bar (*p*<0.05). Treatment legend is given in Figure 1.

**Supplementary Figure S3. Soil water content in different treatments at the rice tillering and heading stage.** Significant differences of different treatments at the same rice growing stage are showed by different letters over error bar (*p*<0.05). Treatment legend is given in Figure 1.

**Supplementary Figure S4. Soil pH values in different treatments at the rice tillering and heading stage.** Significant differences of different treatments at the same rice growing stage are showed by different letters over error bar (*p*<0.05). Treatment legend is given in Figure 1.

**Supplementary Table S1.** The correlation based on Spearman analysis among CH_4_ emission and other observed characteristics.

|  | Methanogenic activity | CH_4_ oxidation activity | MBC | DOC | pH | Water content | 16S rRNA genes of methanogens | *pmoA*  gene |
| --- | --- | --- | --- | --- | --- | --- | --- | --- |
| CH_4_ | .500** | .533** | -.629** | -.293 | .171 | .265 | -.024 | -.0558^*^ |
| Methanogenic activity |  | .515^**^ | -.805^**^ | -.824^**^ | .042 | .095 | .318 | -.305 |
| CH_4_ oxidation activity |  |  | -.600^**^ | -.394^*^ | .439^*^ | .542^**^ | 0.41 | .224 |
| MBC |  |  |  | .586^**^ | -.206 | -.343 | -.241 | .480 |
| DOC |  |  |  |  | -.138 | -.060 | -.518* | -.259 |
| pH |  |  |  |  |  | .888^**^ | -.266 | .097 |
| Water content |  |  |  |  |  |  | -.341 | .174 |
| 16S rRNA genes of methanogens |  |  |  |  |  |  |  | .430 |

**Supplementary Table S2.** Lighting time and temperature settings during the rice growing season.

| Lighting time | Date/Year  2013-2014 |  | 00:01  /°C | 03:00  /°C | 06:00  /°C | 09:00  /°C | 13:00  /°C | 15:00  /°C | 18:00  /°C | 22:00  /°C |
| --- | --- | --- | --- | --- | --- | --- | --- | --- | --- | --- |
| 6:00-2；18:00-0 | 12.6-12.14 | aTem | 15 | 14 | 13 | 17 | 21.5 | 19 | 17 | 15 |
|  |  | eTem | 18 | 17 | 16 | 20 | 24.5 | 22 | 20 | 18 |
| 6:00-2；18:00-0 | 12.15-12.20 | aTem | 15 | 15 | 15 | 19 | 23.5 | 20 | 17 | 15 |
|  |  | eTem | 18 | 18 | 18 | 22 | 26.5 | 23 | 20 | 18 |
| 5:40-2；18:00-0 | 12.21-12.25 | aTem | 15.2 | 15.2 | 17 | 20 | 25.1 | 21 | 18 | 15.2 |
|  |  | eTem | 18.2 | 18.2 | 20 | 23 | 28.1 | 24 | 21 | 18.2 |
| 5:30-2；18:10-0 | 12.26-12.30 | aTem | 18.6 | 18.6 | 20 | 22 | 25.8 | 22 | 20 | 18.6 |
|  |  | eTem | 21.6 | 21.6 | 23 | 25 | 28.8 | 25 | 23 | 21.6 |
| 5:20-2；18:20-0 | 12.31-1.4 | aTem | 19 | 19 | 22 | 25 | 27.9 | 25 | 22 | 19 |
|  |  | eTem | 22 | 22 | 25 | 28 | 30.9 | 28 | 25 | 22 |
| 5:10-2；18:30-0 | 1.5-1.9 | aTem | 19.3 | 19.3 | 23 | 26 | 28.5 | 26 | 23 | 19.3 |
|  |  | eTem | 22.3 | 22.3 | 26 | 29 | 31.5 | 29 | 26 | 22.3 |
| 5:00-2；18:40-0 | 1.10-1.14 | aTem | 20 | 20 | 23.5 | 27 | 29.2 | 27.5 | 24 | 20.2 |
|  |  | eTem | 23 | 23 | 26.5 | 30 | 32.2 | 30.5 | 27 | 23.2 |
| 4:50-2；18:50-0 | 1.15-1.19 | aTem | 20.5 | 20.5 | 24 | 27.5 | 30 | 28 | 24.5 | 20.7 |
|  |  | eTem | 23.5 | 23.5 | 27 | 30.5 | 33 | 31 | 27.5 | 23.7 |
| 4:40-2；19:00-0 | 1.20-1.24 | aTem | 21.2 | 21.2 | 24.5 | 28 | 30.8 | 28.5 | 25 | 21.5 |
|  |  | eTem | 24.2 | 24.2 | 27.5 | 31 | 33.8 | 31.5 | 28 | 24.5 |
| 4:30-2；19:10-0 | 1.25-1.29 | aTem | 22 | 22 | 25.5 | 28.5 | 31.5 | 29 | 25.7 | 22.3 |
|  |  | eTem | 25 | 25 | 28.5 | 31.5 | 34.5 | 32 | 28.7 | 25.3 |
| 4:20-2；19:20-0 | 1.30-2.3 | aTem | 23 | 23 | 26.5 | 29.2 | 32 | 30 | 26.7 | 23.3 |
|  |  | eTem | 26 | 26 | 29.5 | 32.2 | 35 | 33 | 29.7 | 26.3 |
| 4:10-2；19:30-0 | 2.4-2.8 | aTem | 24 | 24 | 27.6 | 30.5 | 32.8 | 31 | 27.9 | 24.3 |
|  |  | eTem | 27 | 27 | 30.6 | 33.5 | 35.8 | 34 | 30.9 | 27.3 |
| 4:00-2；19:35-0 | 2.9-2.13 | aTem | 25 | 25 | 28.6 | 31 | 33.4 | 31.2 | 28.9 | 25.4 |
|  |  | eTem | 28 | 28 | 31.6 | 33 | 36.4 | 34.2 | 31.9 | 28.4 |
| 4:05-2；19:30-0 | 2.14-2.18 | aTem | 25.8 | 25.8 | 29 | 31.2 | 34 | 31.5 | 29.3 | 26.1 |
|  |  | eTem | 28.8 | 28.8 | 32 | 34.2 | 37 | 34.5 | 32.3 | 29.1 |
| 4:10-2；19:25-0 | 2.19-2.23 | aTem | 26.4 | 26.4 | 30.1 | 33 | 35 | 33.2 | 30.2 | 26.7 |
|  |  | eTem | 29.4 | 29.4 | 33.1 | 36 | 38 | 36.2 | 33.2 | 29.7 |
| 4:15-2；19:20-0 | 2.24-2.28 | aTem | 26.8 | 26.8 | 30.3 | 33.2 | 35.4 | 33.5 | 30.5 | 27 |
|  |  | eTem | 29.8 | 29.8 | 33.3 | 36.2 | 38.4 | 36.5 | 33.5 | 30 |
| 4:20-2；19:15-0 | 3.1-3.5 | aTem | 27.2 | 27.2 | 30.5 | 33.5 | 35.7 | 34 | 30.8 | 27.4 |
|  |  | eTem | 30.2 | 30.2 | 33.5 | 36.5 | 38.7 | 37 | 33.8 | 30.4 |
| 4:25-2；19:10-0 | 3.6-3.10 | aTem | 28 | 28 | 31 | 34 | 36.2 | 34.2 | 31.2 | 28.2 |
|  |  | eTem | 31 | 31 | 34 | 37 | 39.2 | 37.2 | 34.2 | 31.2 |
| 4:30-2；19:05-0 | 3.11-3.15 | aTem | 28 | 28 | 31 | 34 | 36.2 | 34.2 | 31.2 | 28.2 |
|  |  | eTem | 31 | 31 | 34 | 37 | 39.2 | 37.2 | 34.2 | 31.2 |
| 4:25-2；19:00-0 | 3.16-3.20 | aTem | 28.2 | 28.2 | 31.5 | 34.6 | 36.5 | 34.6 | 31.6 | 28.5 |
|  |  | eTem | 31.2 | 31.2 | 34.5 | 37.6 | 39.5 | 37.6 | 34.6 | 31.5 |
| 4:25-2；19:00-0 | 3.21-3.25 | aTem | 28 | 28 | 31.2 | 34.3 | 36.2 | 34.3 | 31.2 | 28.2 |
|  |  | eTem | 31 | 31 | 34.2 | 37.3 | 39.2 | 37.3 | 34.2 | 31.2 |
| 4:25-2；19:00-0 | 3.26-3.30 | aTem | 28.4 | 28.4 | 31.5 | 34.6 | 36.5 | 34.6 | 31.5 | 28.6 |
|  |  | eTem | 31.4 | 31.4 | 34.5 | 37.6 | 39.5 | 37.6 | 34.5 | 31.6 |
| 4:25-2；19:00-0 | 3.31-4.4 | aTem | 28.4 | 28.4 | 31.5 | 34.6 | 36.5 | 34.6 | 31.5 | 28.6 |
|  |  | eTem | 31.4 | 31.4 | 34.5 | 37.6 | 39.5 | 37.6 | 34.5 | 31.6 |
| 4:30-2；19:00-0 | 4.5-4.9 | aTem | 28.3 | 28.3 | 31.3 | 34.4 | 36.3 | 34.4 | 31.3 | 28.4 |
|  |  | eTem | 31.3 | 31.3 | 34.3 | 37.4 | 39.3 | 37.4 | 34.3 | 31.4 |
| 4:35-2；19:00-0 | 4.10-4.14 | aTem | 28.6 | 28.6 | 31.6 | 34.7 | 36.6 | 34.7 | 31.6 | 28.7 |
|  |  | eTem | 31.6 | 31.6 | 34.6 | 37.7 | 39.6 | 37.7 | 34.6 | 31.7 |
| 4:35-2；19:00-0 | 4.15-4.19 | aTem | 28.4 | 28.4 | 31.4 | 34.5 | 36.4 | 34.5 | 31.4 | 28.5 |
|  |  | eTem | 31.4 | 31.4 | 34.4 | 37.5 | 39.4 | 37.5 | 34.4 | 31.5 |
| 4:40-2；19:00-0 | 4.20-4.24 | aTem | 28.6 | 28.6 | 31.6 | 34.7 | 36.6 | 34.7 | 31.6 | 28.7 |
|  |  | eTem | 31.6 | 31.6 | 34.6 | 37.7 | 39.6 | 37.7 | 34.6 | 31.7 |
| 4:40-2；19:00-0 | 4.25-4.29 | aTem | 28.6 | 28.6 | 31.6 | 34.7 | 36.6 | 34.7 | 31.6 | 28.7 |
|  |  | eTem | 31.6 | 31.6 | 34.6 | 37.7 | 39.6 | 37.7 | 34.6 | 31.7 |

aTem and eTem represent ambient and elevated temperature respectively. ×:××-2 means lights will be turned on after this time, ×:××-0 means lights will be switched off after this time. Air temperature was set to follow the ambient air temperature of April to August in Hangzhou city, China for the control treatments and +3°C for the elevated temperature treatments.

**Supplementary Table S3.** qPCR primers and thermal cycling conditions

| Gene target:  Primers | Sequences (5’-3’) | Amplicon size (bp) | Thermal conditions | Reference |
| --- | --- | --- | --- | --- |
| 16S rRNA genes of methanogens:  0357f/0691r | CCCTACGGGGCGCAGCAG | 367 | 50 °C | 1 |
|  | GGATTACARGATTTCAC |  |  |  |
| *pmoA*:  A189f/mb661r | GGNGACTGGGACTTCTGG | 510 | 55 °C | 2 |
|  | CCGGMGCAACGTCYTTACC |  |  |  |

**Supplementary Methods**

**CH_4_ flux measurement.** CH_4_ fluxes were measured in the morning between 8:00 and 10:00 every 5 days in rice growing season. A polyvinylchloride-board made chamber (60 cm high and 23 cm inner diameter) equipped with a dry battery driven fan and a gas sampling space tightened with butyl rubber lids was set to be put in the water tank (5 cm high) of the rice pot for gas sampling. Samples of the chamber air were collected by 20 ml syringe at 0, 30 and 60 min after enclosure. Samples were then injected into pre-evacuated 18 ml vials fitted with butyl rubber lids and aluminum crowns for analysis at laboratory. Air temperature inside the chamber was simultaneously measured. The concentration of CH_4_ was analyzed using a gas chromatograph (GC-14B, Shimadzu, Japan)^3^ with a stainless steel column packed with 100-mesh Porapack Q and coupled with a flame ionization detector (FID). The oven and the FID detector were operated at 60 °C and 300 °C, respectively. CH_4_ emission flux was calculated by the equation: F=ρ×h×dc/dt×273/T. F is the CH_4_ emission flux, ρ is the density of CH_4_ at temperature T, h is the height of the closed chamber, dc/dt is the change of the CH_4_ concentration per unit in the closed chamber, and T is the Fahrenheit temperature of the closed chamber. Samples sets were rejected unless they yielded a linear regression value of r^2^ greater than 0.90.

**Determination of MBC and DOC.** Extractable microbial biomass C (MBC) of fresh soil sample was measured by fumigation-extraction method^4^. 5.0 g dry weight equivalent fresh soil sample was fumigated and non-fumigated with CHCl_3_ for 24 h at 28 °C. Then, soluble organic C was extracted by 25 ml 0.5 mol L^-1^ K_2_SO_4_ from fumigated and non-fumigated samples. The content of K_2_SO_4_-extracted C from the CHCl_3_ treated and untreated samples was determined by an automated TOC Analyzer (Multi N/C 2100, Jena, Germany) by using a 750°C combustion technique with non-dispersive infrared detection. MBC was calculated as follows: MBC=EC/k_EC_, where EC= (organic C extracted from fumigated soils) - (organic C extracted from non-fumigated soils), and k_EC_=0.45. The organic C extracted from non-fumigated soils was considered as DOC.

**Supplementary References**

1. Watanabe, T., Asakawa, S., Nakamura, A., Nagaoka, K. & Kimura, M. DGGE method for analyzing 16S rDNA of methanogenic archaeal community in paddy field soil. *FEMS Microbiol. Lett.* **232**, 153-163 (2004).

2. Lüke, C. *et al.* Macroecology of methane-oxidizing bacteria: the β-diversity of *pmoA* genotypes in tropical and subtropical rice paddies. *Environ. Microbiol.* **16**, 72-83 (2014).

3. Dong, D. *et al.* Responses of methane emissions and rice yield to applications of biochar and straw in a paddy field. *J. Soils Sediments* **13**, 1450-1460 (2013).

4. Vance, E., Brookes, P. & Jenkinson, D. An extraction method for measuring soil microbial biomass C. *Soil Biol. Biochem.* **19**, 703-707 (1987).
